# The geo-politics of resilience: On the historical convergence between ecology, artificial intelligence, and corporate strategy

**DOI:** 10.1177/14614448251336420

**Published:** 2025-08-02

**Authors:** Orit Halpern

**Affiliations:** Technische Universität Dresden, Germany

**Keywords:** Artificial intelligence, cybernetics, decolonization, digital twins, ecology, energy, environmental management, logistics, neoliberalism, race and population, resilience, shell oil scenario planning

## Abstract

Today, few terms are more central to policy, planning, or economics than the term “resilience.” From urban planning to twining the earth systems, we have come to understand systems as constantly in a state of crisis that needs perpetual management. This article traces the rise of resilience as a dominant epistemology and practice in environmental management, logistics, demography, and energy. I will argue that resilience has become the dominant discourse by which time and uncertainty are currently being managed in the wake of post-World War II decolonization, generating new techniques such as digital twinning and generative artificial intelligence (AI). Moreover, resilience has become a new logic making the planet, and its living populations, computationally measurable and representable, and amenable to new forms of technical manipulation and action.

*Even when handled well, strategic change can be extremely disruptive to organizations . . . marrying gen AI with digital twins can produce spectacular outcomes.*

—Kenny and Ganna Pogrebena, *Harvard Business Review*, 23 September 2023

*The climate crisis, the pandemic, regional conflicts and the ensuing refugee crisis, are reminders of the unprecedented capacity of external shocks to disrupt economies and societies. These shocks – whether environmental, economic, geopolitical, or health-related – are only increasing in frequency and severity, making strengthening resilience an imperative to ensure sustainable and inclusive growth in the future.*

—World Economic Forum, Resilience Consortium, 2022

*To seize opportunities in an evolving global order, leaders will need to build geopolitical resilience by investing in three domains: insight, oversight, and foresight.*

—McKinsey & Company, Building Geo-political Resilience: The People Agenda, 17 December 2024

Few terms have risen to greater prominence in the past decade than “resilience.” There is a widespread consensus among corporate managers, policy makers, politicians, and planners that the future is full of disruption. For example, in 2019, the European Union (EU) embarked on one of the most expansive visions of managing the planet through computing to date—the EU-funded Destination Earth (DestinE) project. The project aims to model the earth system with never-before-seen scales of data granularity. No longer a simulation, this is the so-called “digital twins of the earth.” Digital twins of the earth aim to create continuous, real-time data flows between various kinds of environmentally situated sensors and other sources of data, on one hand, and high-capacity computing facilities that employ AI applications, on the other. The project imagines linking artificial intelligence (AI) with GIS systems, citizen sensing projects, government data, and a myriad of other systems monitoring temperature, pollution, and biodiversity about both human activities and natural systems to

develop a highly-accurate digital model of the Earth (a digital twin of the Earth) to model, monitor and simulate natural phenomena, hazards and the related human activities. These groundbreaking features assist users in designing accurate and actionable adaptation strategies and mitigation measures. (EU, 2024)

What is worth noting is that in the case of Destination Earth, the implicit assumption is that disasters cannot be avoided; they can only be “mitigated.” DestinE is funded by the Recovery and Resilience Fund of the EU. The language of adaptation, evolution, and constant change suggests that digital technology and biological survival are now viewed as both contiguous and mutually reinforcing. This is part of a broader narrative often used to reify and justify increased expenditure in AI and computing involving the language of resilience.

The EU is not alone in addressing the climate and social crisis through AI and twins. Corporations marketing ubiquitous computing infrastructures such as Huawei urge a future of generative infrastructures for a rapidly urbanizing planet. “Resilient cities,” they argue, “[demand that] the next step in this urban evolution will be ‘cognitive’ cities powered by AI. Making this happen will require a host of technologies, including one that’s already being adopted by forward-looking cities: the digital twin” (Nazir, 2024). A recent 2024 Gartner hype cycle report on supply chain technologies, reiterated the trope that resilience through AI and twinning is central for corporate competitiveness:

Supply chain planning (SCP) plays a role in improving uncertainty management. This means using technology [digital twins and AI] to change the state of the supply chain from fragile, through resilient . . . a top theme for this year’s Top 25 supply chains. (Joe Graham, 2024)^1^

Generativity and cognition, but no longer solely human, are key to this concept of evolution and survival through technology. Nvidia’s Omniverse platform services many industries from molecular biology to auto manufacturing. Central to their business model is the use of AI and 3D virtual reality environments (sometimes labeled twins) to optimize supply chains, environmental management, automated design, and manufacturing. Automated data collection, analytics, and machine learning, to quote Jensen Huang of Nvidia, “automate inference,” and insert it through the cloud and Internet of things into the environment (Goode, 2024). This AI abetted inference, according to Huang, is the key to “never before seen” predictions, and a necessity for industries and researchers wishing to retain competitiveness, advanced cybersecurity, and foster innovation (Ord, 2024).

The idea that decision-making at scale will be embedded into the environment and will be the source of profit and security is key. Engineers and corporations label this “infrastructure as service” an extension of the model of “software as service” and of cloud computing as service that has dominated computing since the early 2000s. For example, in cases like DestinE the function of the twin is also to train machine learning algorithms. The infrastructure for data collection and visualization, and ultimately the lives and environments documented, become assets as part of a service to other organizations seeking to develop new technologies.

These cases demonstrate the vivid imagination of ubiquitous computing, smart infrastructure, and AI in managing planetary, and soon extra-planetary, crisis of environment and politics. In this article, I will investigate the relationship between platform technologies such as digital twins and histories of energy and ecology to mark the rise of what I am labeling *the geo-politics of resilience*, a new form of capitalism and politics grounded in AI and big data and marked by transformations of life itself into a service infrastructure for computing.

Although there have long been calls from governments and corporations to adopt digital strategies for global competitiveness; the current discourse of resilience has a unique relation to generative AI, biological evolution, and the fantasy of twins and ubiquitous computing infrastructures. This is an imaginary that envisions infrastructures as “cognitive” and planetary, cognitive in the sense capable of autonomous decision-making at scale. Planetary implying here that the forms of territory being managed no longer subscribe to older models of the nation-state, and bridge different forms of materiality—cognitive, biological, and inorganic—and different populations—human, more than human, machine. Moreover, computing takes on imagined biological and survivalist capacities. Resilience, as the opening quotes suggest, relies on the automation and normalization of shock, not as an unusual event demanding structural readjustment, but as an ongoing condition that assumes readjustment itself as technically automatable and embedded *into* infrastructure, what I am labeling “life as service.” Computing, crisis, and evolutionary biology are unified making artificially intelligent platforms seemingly natural and *necessary* solutions to a world now understood as constantly facing shock and catastrophe and an economy no longer understood to be growing teleologically.

I suggest that in many ways resilience is a mandate. All systems *must* be made resilient to survive—whether democracy or supply chains. This is in reference to my own work with Robert Mitchell in *The Smartness Mandate* (Halpern and Mitchell, 2023).^2^ Initially, resilience appeared to be something smartness produces, an observation I reiterate here. But the events of recent years, such as the unremitting rise of the alt-right, the direct new relationship between silicon valley and anti-democratic, autocratic, and neo-Malthusian figures such as Elon Musk and Peter Thiel, demand revisiting resilience.^3^ Smartness’ cyborgian promises of new relations to the environment and each other, what we refer to as the “biopolitical learning consensus,” a hopeful aspiration that through smart and artificially intelligent technologies new forms of learning from populations might generate non-extremist, less extractionary, equitous, and perhaps diverse political economies, appear naïve now (Halpern and Mitchell, 2023). In the present, the replacement of the language of smartness by twinning, artificiality, and resilience, suggests that something is happening. This article is grounded in a fundamental effort to extend this earlier work on resilience and smartness with a renewed focus on the neo-Malthusian, (post)-colonial, and geopolitical elements of a history of ubiquitous computing. In this focus, I follow the lead of the many scholars such as Ginger Nolan, Anna Tsing, Ned Rossiter, Charmaine Chua, and Deborah Cowan who have explicitly examined how coloniality, global economies, and contemporary digital infrastructures intersect (Chua et al., 2018; Cowan, 2014; Nolan, 2021; Rossiter, 2016; Tsing, 2004).

This marriage of computation and governmentality poses questions. What are the emerging images or *representations* of the planet that attend resilience? What are the forms of *territorialization* engendered through practices such as twinning? Territorialization refers to how economic and political actors colonize and control time and space through future-oriented activities such as investment, speculation, and prospecting (Andersson, 2020). What are the strategies and practices of resilience; how did *adaptive management* come to dominate ideas of planning and prediction? Finally, what are the ideological underpinnings and planetary *imaginaries* of resilience? In short, how have histories of modeling nature become technologies that we have become *conditioned* to use every day *without question*. And moreover, have become dependent upon, perhaps, for survival.

In order to begin answering these questions, this essay will trace a history of computational efforts to model the planet’s population and ecosystems, and corporate strategies to manage risk in the face of the geo-political turmoil, particularly the OPEC oil crisis, of the 1970s. Putting contemporary AI, and particularly interfaces and attention platforms such as digital twins, within histories of managing both the environmental and the energy crisis that came to consciousness at that time makes visible the neo-colonial and extractive histories that inform the technology. While I am hardly the first to demarcate this time period as central to rise of the information economy, financialization, globalization, and environmentalism (see, for example, Beck, 1992; Harvey, 1990; Sassen, 2001), I want to connect these histories with AI, and with the material infrastructures of computing, with twinning as an exemplary use case. While this special issue is aimed at digital twins as an object, the hope here is to situate current discourses of twinning within historical context, and to ask about the political economies and ideologies of twinning as part of a larger governmental logic of AI.

## Limits into growth

The early 1970s inaugurated two discourses of environment and economy that continue to inform contemporary digital infrastructures and information economies: one of limits and one of resilience. The two are interrelated but possess some considerable differences in their models of nature, information, and technology.

In 1971, the Chicago School neoliberal economist Milton Friedman, in response to the petrodollar crisis and the end of Bretton Woods, announced a “major need for a broad, widely based, active, and *resilient* futures market” (Friedman, 2011). Counter to standard understandings of the economy at the time, he projected a positive valence for active, volatile markets. For Friedman, the collapse of the Gold Standard and its model of economic stability was not a calamity, but an opportunity for creating a new, what he labeled “resilient” system of international currency exchange. Friedman argued that the solution could not be another rigid centrally-controlled system, but instead a futures market for currencies: that is, a system that might allow those engaged in foreign trade to hedge the risks associated with currency exchange changes. That system would become one of futures exchanges and new computational technologies—such as derivative pricing equations. These equations automated decision-making and facilitated betting and value extraction not from the value of an asset but from the variation *between* assets. As historian Caleb Wellum (2020: 9) relates, “The president of the Petroleum Associates of the NYCE told the Wall Street Journal in 1974, ‘futures trading doesn’t work in a stable market,’ but it could help to ‘flatten out the peaks and the rises in the unstable market that the oil embargo had created.’” Futures markets were technologies of managing a future that was *not predictable*.

To paraphrase Milton Friedman, “[economic models] are engines not cameras”(MacKenzie, 2006). They do not represent worlds; they make them. Perhaps the ultimate in virtual avatars? The models underpinning futures markets made the market and facilitated the continuation of carbon-based economies by transforming the limits to energy into speculative futures. Futures markets and particularly the derivative trading instruments emerging from this period, such as the Black Scholes Model, have entered contemporary narratives of “free markets” as the agents that stabilized energy prices, supposedly demonstrating the power of neo-liberal policies and markets to manage geo-political instability over regulatory or state actions and the Carter administration (Wellum, 2020).

Resilience also appeared in another form, seemingly unrelated, shortly thereafter. In 1973, the Canadian ecologist C. S. Holling (1973) introduced a new idea of nature, adaptation, and perhaps even, evolution:

INDIVIDUALS DIE, POPULATIONS DISAPPEAR, and species become extinct. That is one view of the world. But another view of the world concentrates not so much on presence or absence as upon the numbers of organisms and the degree of constancy of their numbers. These are two very different ways of viewing the behavior of systems and the usefulness of the view depends very much on the properties of those concerned. (p. 1)

For Holling, disruption was a regular event. Extinction happens all the time, and environments change. The assumption that systems return to the past was in this model, impossible.

In turn, ecologists had to shift focus away from on the event of extinction, or the numbers of animals or humans in an ecosystem. Rather, Holling suggests, they should focus on the *relationships* in a system. These relationships came to be seen as applications or services that could be maintained irrespective of the life or death of specific populations. These ideas and techniques would by the late 1970s and early 1980s be rebranded as “adaptive management” (Holling, 1978a).

If Friedman’s markets were intimately linked to the rise of computing for calculating derivative instruments, Hollings vision of nature was also tied to digital media. New GIS systems had permitted gathering data and visualizing ecological data across territories and in new ways (Holling, 1978c).

While the goals might be radically different in objective (although both the ecologist managing forests and the neo-liberal had economic concerns), both posit a world model of constant volatility and instability and urge new calculative techniques to manage uncertainty/

If resilience suggested a world of constant volatility to be managed through new calculative techniques, then its doppelganger was the *Limits to Growth Report* issued in 1972. The widely heralded report is often regarded as the start of the contemporary environmental movement and might also be understood as progenitor for systems such as DestinE that seek to model not only climate, but social phenomena (Laakkonen et al., 2016).

The result of 2 years of computerized data analysis and modeling, using a world model labeled World 3, the report predicted the collapse of the global world system (Meadows et al., 1972: 23)^4^. This future was not inevitable. The authors urged, “It is possible to alter these growth trends [the disturbances human activities have created to the ecosystem] and to establish a condition of ecological and economic stability that is sustainable far into the future” (Meadows et al., 1972). The model posited human behavior and society as “problems” and assumed a stable system that should be returned to. The report was part of a broader framework, as Sabine Höhler has demonstrated, posing the planet as being in crisis, but also planets as being scientifically and technically controllable (Höhler, 2015).

*Limits to Growth* thus forwarded a broader concept of rationality and governance in the Cold War. As Paul Erickson and his colleagues have argued, the Cold War championed a model of rationality that was algorithmic, repeatable, quantitative, and closely correlated with expertise grounded in cybernetics, game theory, and operations research (Erickson et al., 2015). While telling a narrative of demise, the project presented the world as *representable*, amenable to computational modeling and analytics, and technocratically controllable; it *is*, as the report stated, possible to alter the results with planning and scientific knowledge (Meadows et al., 1972: 24; Meadows, 2007). These assumptions about predictability and the mechanical nature of “nature” also ingrained into the media and algorithms of system analysis, all of which worked on models of linear causality (Höhler, 2015).

These two epistemologies were critically linked in supporting a vision that collapsed social systems with biological and earth ones while also assuming there were managerial strategies that might deal with global, and now planetary, problems. However, one (limits) might be understood as assuming the centrality of conscious planning and prediction and the possibility of perfect information, and another worldview imagining a network-driven (perhaps market-driven) decision-making that never predicts the future but adapts to it. If *Limits* defined a “closed world” with finite resources, *resilience* defined an eternally dynamic evolving system where resource limits become frontiers. Limits to growth or limits *into* growth.

## The knowability of the future?

Another way to read these debates about the nature of nature and human societies is as positing contesting visions of technology and determinism. Almost immediately, neoliberal economists attacked the report. In 1974, in his Nobel Prize for Economics speech, the neoliberal economist Friedrich Hayek openly disparaged the idea of predictive modeling in *Limits*. For Hayek, world models were authoritarian models; they violated the possibility that free individuals networked into markets might act in innovative ways. *Limits*, he argued, relied on the assumption that these scientists possessed perfect information, and that the world never changed. This was part of a more general plea, addressed to both mainstream economists and their leftist critics, for a more modest epistemology that would give up on the dream of complete control over the future (Hayek, 1974).

Neoliberal economists were not alone in this debate over prediction, modeling, and stewardship of population. Economists (ironically perhaps) from the International Monetary Fund almost immediately noted the assumptions of the report favored the Global North by assuming that change was impossible. Mahbub ul-Haq (1972), prominent Pakistani economist and inventor of the Human Development Index, stated,

In an age of the most dramatic technological progress, the authors contend that there cannot be a continuation of such rapid progress in the future. . . The model *assumes* that certain things in this world – population, capital stock, pollution – will grow at exponential rates; but it *assumes* that certain other things – specifically technology to enlarge the resource base and to fight pollution – will not grow exponentially. . . The basic weakness of the *Limits to Growth* thesis is not so much that it is alarmist but that it is complacent. It is alarmist about the physical limits which may in practice be extended by continued technological progress, but complacent about the social and political problems which its own prescriptions would only exacerbate. . . For the developing world, however, zero growth offers only a prospect of despair and world income redistribution is merely a wistful dream.

At stake in this discussion was learning—don’t humans learn? And don’t they innovate? And about biological determinism? Are certain humans naturally ordained to horrible fates and incapable of changing? If there is technology, can this lead to wealth redistribution? Haq implied that behind the call to attend to the environment and pollution, there also lay a neo-Malthusian call to control population (only in the Global South) in possibly eugenic manners paraded under the umbrella of the carrying capacity of the planet. The question emerged: what technologies might change limits into growth? And for whom? And finally, how should information be processed at scale to make the optimal (however that is defined) decisions about wealth or resource allocations? These discourses were part of a broader developmentalist discourse emerging that problematically paralleled technology and economy with equity and progress (Dirlik, 2012). As the two critiques demonstrate, a central animating concern with technology for global governance is the relationship between determinism, information, and biology.

## Becoming resilient

Haq’s critique suggests that the idea of a perfectly calculatable, but unable to adapt or learn, planet came at odds with transforming geo-political realities. While economists, developmentalists, and politicians from the Global South condemned the report, one unlikely site of both critique and technical innovation came from ecologists, who were among the first to take up the challenge of volatility and change in complex systems.

**Figure 1.**
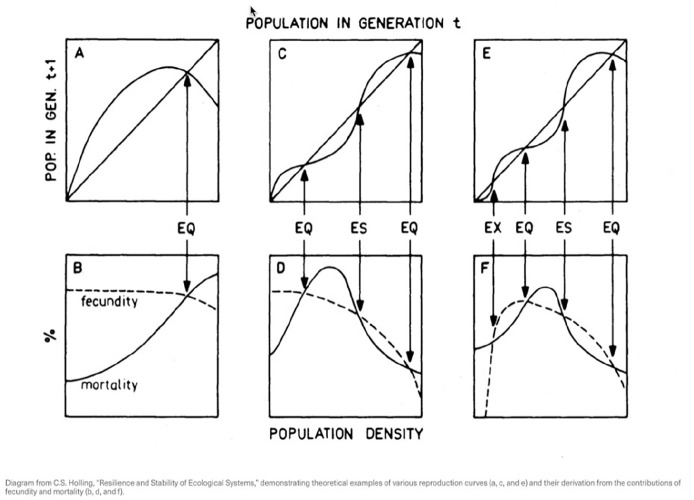
Future Population Projections from “Resilience and Stability in Ecological Systems” (Holling, 1973).

Offering an alternative to the seemingly linear models of *Limits to Growth*, the Canadian ecologist C. S. Holling developed the concept of resilience to *contest the premise* that ecosystems were most healthy when they returned quickly to an equilibrium state after being disturbed (Figure 1). While openly supportive of the conservation goals of *Limits* and the use of quantitative models, Holling also opposes the use of linear and deterministic algorithmic models to simulate nature, and suggests that ecomanagement might be reformulated as a problem in information processing and redefining data (from quantitative to qualitative) (Holling, 1978b: 6).

Returning to the 1973 paper, *Resilience and Stability in Ecological Systems*, Holling (1973: 1–23) noted,

. . . if we are dealing with a system profoundly affected by changes external to it, and *continually confronted by the unexpected, the constancy of its behavior becomes less important than the persistence of the relationships*^5^. Attention shifts, therefore, to the *qualitative*. . .

Holling (1978b: 6) argues again that it is the “persistence of relationships” that must be studied and argues quantitative and qualitative measures are both necessary (Cooper and Walker, 2011; Lindseth, 2011). Qualitative does not denote non-quantitative as much as non-discrete and non-static. The key idea being that complexity and uncertainty in managing systems demands new methods and new relationships to data.

**Figure 2.**
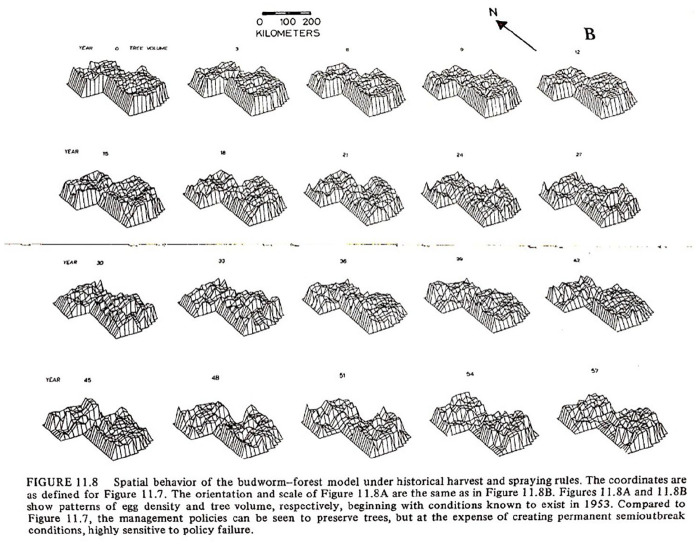
Topological models generated from historical data since 1951 of budworm population densities in space (Holling, 1978c: 164).

In turn, managers should focus on *processes* that define a system. For example, in the boreal forest (Figure 2), the absolute number of spruces is not important; what is important is the ability for the forest to rejuvenate and continue growing trees. This regenerative capacity depends on fluctuating numbers of populations between spruce, fir, birch, and budworms. What is key is that the regeneration demands a diversity of species. These maps visualize changes in population densities between different trees and insects. The term “environmental services,” later (in the 1980s) “ecosystem services,” was created to describe this process of identifying and managing processes rather than static numbers (Braat and de Groot, 2012). This language in focusing on relations between agents in a system also bears significant similarity to contemporary ideas of infrastructures as resources or services.

## Visualization and territorialization

This view of nature is also grounded in media. The visualization made above (Figure 2) was among the first to use the Canadian Geographic System (CGIS), often considered one of the roots of contemporary GIS, in the early 1970s. Without new forms of data collection, computer visualization, and satellite infrastructure, such visions of the environment would not be possible (Holling, 1978c: 164).

These ecology studies also introduced computer simulation into ecology. In a following study Holling and his colleagues explained that

This paper demonstrates that by making reasonable and easily verifiable assumptions . . . it is possible to produce an *effective algorithm* for *forest policy evaluation*. The method proposed and applied to the case of the New Brunswick forests consists of: (1) construction of a forest *simulation* to generate policy contingent distributions of outcomes, and (2) employment of *stochastic* dominance to identify a nondominated set of policies.^6^

By combining field-based data, GIS information, and mathematical models, the teams sought to produce new automated and algorithmic ways to deal with constantly varying systems, and produce simulations through computer automation to predict complex ecosystem behavior (Ludwig et al., 1978: 315).^7^

## Adaptive management and the epistemology of ignorance

The drive to merge the quantitative and qualitative data and to develop new modes of simulation and territorialization was linked to a worldview shared by both industry and ecology of the future as uncertain and un-representable. As Holling (1973) notes, “Flowing from this would be not the presumption of sufficient knowledge, but the recognition of our ignorance: not the assumption that future events are expected, but that they will be unexpected.” In short, *expect the unexpected*. Plan for extreme events without any conception of absolute prediction and assuming that the past does not linearly predict the future.

The idea of a generative and responsive model of managing nature is integral to the fantasy of adaptive management. Digital twinning in this case becomes a central technology because of the belief that twins, unlike static models, deal with stochastic processes and uncertainty at scale and in real time. For example, the NASA Apollo moon missions that took the image of earthrise and supposedly inspired the *Limits to Growth* also saw the emergence of the language of digital “twins.” NASA engineers working on Apollo 8 coined the term “twin” to argue for a cybernetic vision of simulation, where the virtual model would be tied to the “real” model in continuous loops of real-time data exchange (Grieves and Vickers, 2017; Lawton, 2023). According to leaders at NASA, recounting this history much later (and its key that this language is from 2021 not 1968), what differentiated digital twins from the simulation technologies was “scale, ordinality, and non-deterministic nature of models” (Allen, 2021). That is to say that twins, unlike other algorithmic or simulation technologies, were key because they could address new scales—planetary modeling, testing of phenomena impossible to trial within a laboratory setting, visualization of complex systems—and temporalities—stochastic, unpredictable futures, complexity. Twins were imagined as a technology built for the unforeseen accident or disaster. Such twins are credited with assisting in the rescue of the failed Apollo 13 mission (Allen, 2021).

Through organizations such as the Stockholm Resilience Institute, these ideas spread to business schools, organizational management fields, and psychology. The models of adaptive management and the tactics of systems theory and computation were also creating a new digital and professional field of logistics throughout the 1970s and 1980s (Smith, 2022: 73–76). In the early 2000s in the wake of 9/11 and later the COVID pandemic, resilience and adaptive management has become ubiquitous in the discourse of management consulting firms implementing digital transition strategies. Resilience and its affiliated managerial concepts and practices have now become central to many industries and have increasingly also been rebranded as “change management” in the EU (Pettit et al., 2019).

Resilient ecology and economy must also be seen within a wider context where corporations were actively looking to hedge risk. The same period saw corporations embrace a plethora of new technologies, particularly computers, logistics (the emergence of the bar code and container in shipping as technologies in the late 1960s and 1970s), futures markets in commodities and financial instruments, and total cost analysis to manage what Charmaine Chua and her colleagues have labeled “turbulent circulation”(Chua et al., 2018; Cowan, 2011; LeCavalier, 2016). Corporations thus embraced derivative and options markets to manage the rapid fluctuations in costs due to decolonization and geo-political disruptions of supply chains and access to resources while also transforming their supply chains through new forms of accounting (total cost) that permitted the derivation of value not only from sales, but along the chain and made transport and logistics a site of producing profit (Bernes, 2013). The key feature is how new technologies of hedging risk were naturalized through discourses of resilience, and in turn produced ideas of a world constantly in crisis and demanding adaptive management . . . now increasingly automated.

One critical divergence between business management and eco-management, however, concerns attitudes to diversity and evolution. While adaptive management suggests constant evolution, the question of repetition versus change continues to plague managers. The future, for ecologists, is only made possible if a system is not so perfectly adapted to its present that it cannot evolve. Therefore, biodiversity is necessary for resilience. As Holling and his colleagues stated, “ecological resilience is generated by diverse, but overlapping, function within a scale and by apparently redundant species that operate at different scales, thereby reinforcing function across scales” (Peterson et al., 1998: 6). Perfect optimization would be against the possibility of adaptation which demands redundancy and excess. The key question being what constitutes functionality, and whether different species playing the same role constitute diversity? When extrapolated into social and economic systems, the question became about how change or transition is imagined. And what would it entail? The seemingly oxymoronic idea of automating change seemed to defy the laws of nature, where change demands excessive functionality that cannot be anticipated or replicated, and comes into play under conditions that cannot be forecast.

## Automating adaptation

In our present, these underlying principles of adaptive management and resilience have now become *automated*. Here, the world model of AI and the world model of nature combine.

Returning to DestinE, the project continues to struggle with the contradiction of uncertainty versus prediction. The planet has become a “problem” because of a climate crisis that is supposedly too complex to easily resolve or understand. At the same time, the planet can still be visualized, sensed, and governed through instruments of policy, technology, and the public democratic bodies of the EU. This is a feature that many other twinning projects do not share (i.e. the emphasis on open and democratic governance).

However, the very use of planetary data to train AI in the project demonstrates an epistemology of uncertainty and resilience. This logic enacts itself in Nvidia’s Earth-2 and ForCastNet and most recently Google’s GenCast. Both Google and Nvidia are currently building simulations, maybe twins, to forecast weather *without* the use of physics models, that is, without representation, through pure inductive logic. Both have attained considerable success at micro-forecasts simply using historical data (albeit always collected by EUMETSAT) (Broad, 2024). This imaginary of non-numeric forecasting suggests an epistemic change from predictive modeling to zero-shot learning, and a new concept of generative management of decision-making through automation rather than expertise and scientific reason

**Figure 3.**
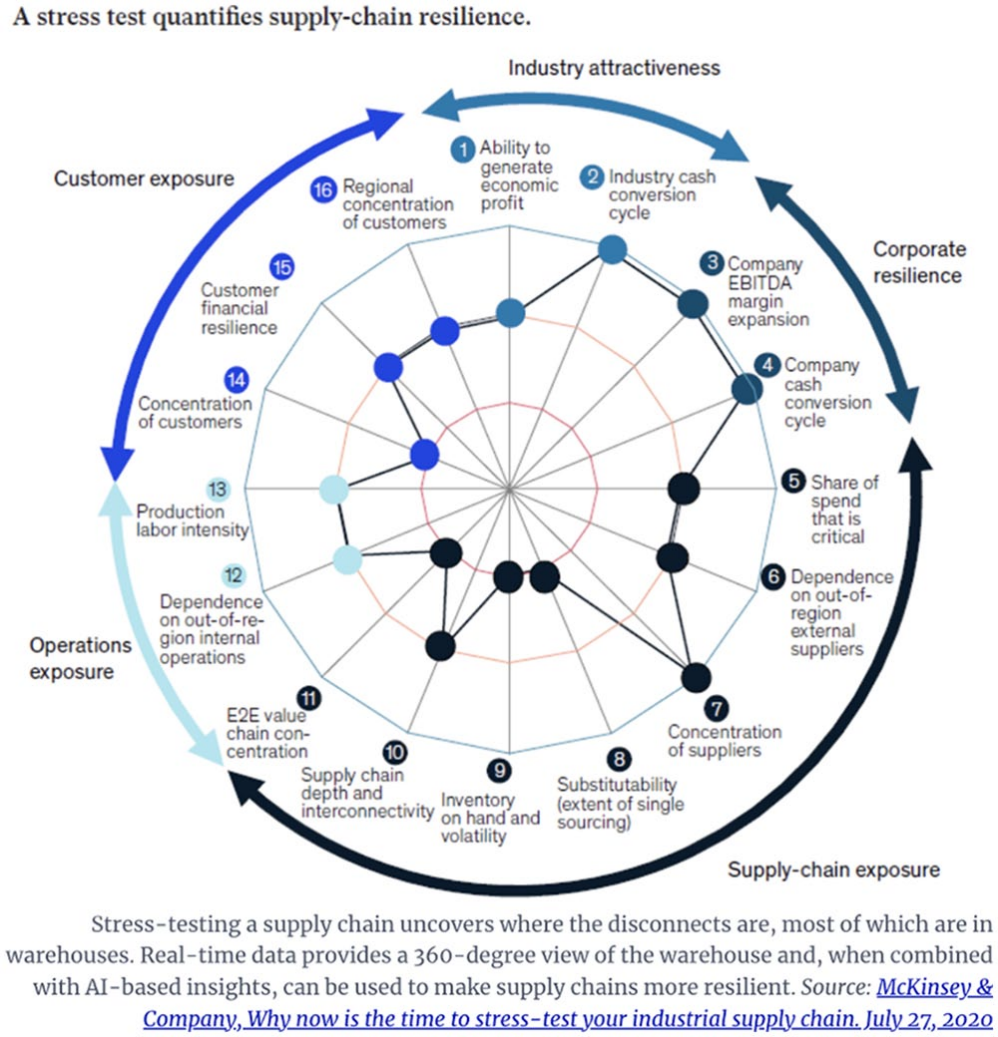
EPM Models from McKinsey & Co. 2020.

Climate modeling is not the only site of automating decision-making at scale. A recent pitch for integrating real-time data into an enterprise management platform to make supply-chains resilient is illustrative. A close study of this image of an automated enterprise management platform with twinning capacity marketed to provide “resilience” demonstrates further these concepts. The platform combines application performance management into site operations across global operations. This combination of data analytics and decision-making facilitates the real-time mapping of the supply chain, cross-organizational data collection, identification of warehouses performing against plan, identification of bottlenecks before they impact performance, evaluation of excess dependence on single vendors, and integration of legacy warehouse management systems (See Figure 3). In theory, this will lead to flexibility, optimization, and innovation across the chain, and will allow the company to manage disruptions—such as trade wars, climate events, and so forth.

Nvidia, Oracle, SAP, IBM, Anaplan, OneStream Software, and Honeywell Connected Warehouse, all offer such EPM platforms today (Columbus, 2022).

The implication being that machines can now be adaptive managers, and through constant reflexive analysis of changing data, modify behavior, and isolate “change processes.” That change itself is now considered something that can be derived from a system is a central value proposition in AI. The uncertainty of the future becoming a frontier for growth in a world understood as resource constrained. As McKinsey & Company suggest, their services entail “future proofing” organizations through becoming “resilient” and “adaptable.” They elaborate, “We don’t just ‘bounce back’ from difficult situations—we ‘bounce forward’ into new realms, learning to be more adaptable as our circumstances evolve and change” (McKinsey & Company, 2021). The language of resilience thus analogizes the corporation to a biological organism, rather than a conscious and intentional actor. The generative and always evolutionary organization.

## Cognitive infrastructures

Accompanying ideas of resilience is an idea not only of managing change itself, but to repeat the words of Jensen Huang, to “automate inference,” or in the language of Huawei, build “cognitive infrastructures.” They imply infrastructures that can adapt to any situation, perhaps autonomously, or at least to guide human flawed decision-making. The marriage of algorithmic decision-making with concepts of de-risking large scale complex systems linked resilience back to finance and eventually to contemporary efforts to automate and manage systems through technologies such as digital twins and big data simulations.

The idea of actually producing cognitive infrastructure as a solution to geo-political impasses and the limits to human reason is implicit in the paradigm of resilience (managers can’t know the future) but found material instantiation in Shell scenario planning, a feature prominently mentioned in virtually every digital and twin platform I researched (Deloitte, 2023; Union, 2024). Shell scenarios were part of the broader turn to logistics and energy future markets previously mentioned. But Shell also introduced the questions of how interfaces serve to condition and introduce managers into the system. They were pioneering in suggesting what Nvidia has now turned into a business model with the Omniverse platform for building simulations,

Shell scenarios were grounded in a new assumption at the time. As Pierre Wack (1985b: 73), a CEO at Shell and the main driver behind scenarios at the company, summated, “The future is no longer stable; it has become a moving target. No single ‘right’ projection can be deduced from past behavior.” Therefore, futures in plural had to be developed, and uncertainty had to be considered.

The function was strategic and psychological. As Wack explained to the *Harvard Business Review* in the mid-1980s,

Scenarios deal with two worlds: the world of facts and the world of perceptions. They explore for facts, but they aim at perceptions inside the heads of decision makers. This transformation process is not trivial—often it does not happen. When it works, it is a creative experience. . .

Wack (1985a) prefaces this with understanding scenarios as “interfaces,” to which he added, “By interface, I mean the point at which the scenario really touches a chord in the manager’s mind—the moment at which it has real meaning for him or her.” Much like the strategies of adaptive management, corporate managers were trained to accept uncertainty. The scenario plans were about retraining executives to imagine a world they could not predict by merging real-world data, computer projection, and fictions. But the practice was itself a medium for cognitive re-conditioning and data collection, creating a feedback loop for collecting manager perception and reformulating it to modify behavior of the organization, analogous to the now computer-automated example of the EPM system offered by McKinsey & Company.

While the practice was inspired by Herman Kahn originally at RAND corporation where systems theories and operations research were being used to project futures, in reality Shell’s ambitions were to monetize uncertainty itself.^8^ The future could no longer be predicted because the newfound sovereignty of the Gulf States and other oil exporting nations in the Global South had found expression through OPEC and the control of oil supplies to the West. Concurrently, the emergence of an environmental movement and an increased possible regulatory environment for energy, as well as geological concepts of resource depletion, all faced Shell with dilemmas as to how to re-invent its business strategy (Andersson, 2020; Mitchell, 2013).

In turn, Shell resolved to deal with this situation, not by responding to the world, but through making the world it sought. Counter to much corporate history, scenarios were not about becoming a “responsible” global citizen or “greening”(Andersson, 2020). Rather, they were about producing a new psychology and new type of organization that could benefit from these adversities. Wack spoke of his intent to create managers capable of accepting uncertainty and experts in adaptation, framing the corporation in terms of evolution and biology. This tendency to analogize through biology was accelerated by the popularization of Shell scenarios through the Global Business Network, a group started by Wack, meeting at the Santa Fe Institute, with enormous impact on the emerging Silicon Valley culture. Stewart Brand openly espoused the idea that scenarios were a way to learn in an “evolutionary system.” As both historians Fred Turner and Jenny Andersson argue, the Santa Fe institute forwarded the idea that corporations were biological organisms driving evolution(Andersson, 2020: 747; Turner, 2006).

**Figure 4.**
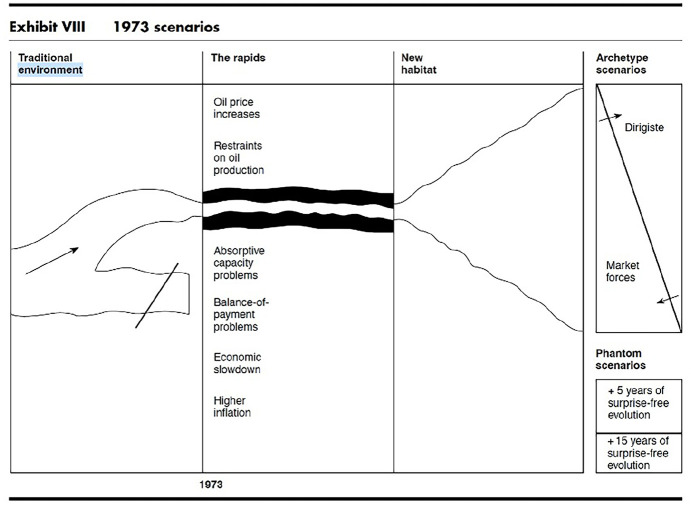
Pierre Wack Scenario Planning Diagram from Harvard Business Review *Uncharted Waters*, 1985, p. 86.

Wack himself suggested through the scenario that the corporation might act like a natural system, thus circumventing the political economic realities of oil extraction and demands for sovereignty coming from the Global South. The diagrams Wack drew replicated those of (see Figure 4) Holling in deploying languages of environment, habitat, and transformation, articulating a new vision of constant flux defined in the language of evolution and adaptation rather than that of geo-politics or political-economy. In fact, the diagram is a “landscape” not an organizational chart.

Wack was, of course, directly engaged with and confronting (or subsuming) ideas of sustainability, environmental management, and limits to resources and an environmental crisis of both climate change and biodiversity, but to different ends. For Shell, managing the limits to growth, environment, and colonialism came with a demand for increased extraction, profit, and unlimited energy. In turn, the corporation turned to creating futures (that they desired).

Interestingly, both the corporate simulations and adaptive management for resilience agreed on the same language and models, suggesting a shared epistemology even if to different ends. Both also turned to technological solutions—management strategies and stochastic thinking—that are arguably now also central to the concept of automating cognition and generativity so vital to AI evangelists.

Above all, corporate pundits of scenario planning repeat that scenarios have a cognitive element. Scenarios are less about precise modeling, and more about changing managerial attitudes and conditioning managers psychologically for uncertainty and change, a theme that often returns in our present with digital simulations and twins. While the capacity of twins to effect change is often in question and difficult to prove (Attaran and Celik, 2023), there is little question that the introduction of a metaverse and gamification to infrastructure management in city planning, environmental management, and industry is training people in both new forms of perception, and learning from users (Huffard, 2023), and/or automating cognition to paraphrase the corporate lingo of both Nvidia and Huawei, therefore the centrality of 3D modeling to twins. For machines, of course, who do not “see” like humans, such renderings often serve little function (except as a training set in cases like autonomous driving), the expenditure of a vast amount of processing power to visibly represent and replicate an earth system or a factory must be understood for human cognition and management, not for the AI supposedly learning and automating processes, and is part of integrating humans into the loop. In fact, both scenarios and metaverses are about making human psychology and machine learning commensurate, and legible to each other.

Shell scenario planning largely replicated the practices suggested by adaptive management proponents but to new ends—the production of cognitive infrastructure (the systematization and automation of human problem solving)—and the biologization and naturalization of the corporation. Popular in Silicon Valley, the Shell scenario deployed the language of adaptation, evolution, and uncertainty to sell the idea that innovation itself could be automated. In a famous video describing scenarios, Wack says that “originality” is the greatest problem in management. Top managers love their own ideas, and fail to figure out the patterns that are actually necessary to understand for invention (Seminar, 1993).

Interestingly, Shell scenario plans were conceived to avoid the problems of simplistic computer prediction, therefore suggesting the broader logic of contemporary simulations. Scenarios sought to produce uncertainty and generativity into the planning process. This contradiction of both seeking to predict the future and supposedly embracing uncertainty is also one of the central discourses in contemporary AI twinning systems to which contemporary organizations suggest that the generative ability of AI to produce unexpected results and the general capacity of systems to constantly “learn” are the solutions for a turbulent planet.

## Generative territories

In our present, contemporary AI infrastructures are the inheritors of these models of managing politics and geography through technical solutions that balance impossible demands—the limits to human reason, resources, even environmental carrying capacities with the imaginary of speculative growth for capital. For example, the Honeywell Corporation provides digital twins and asset performance models to Norway for use on the North Sea oil platforms that provide energy to Norway and the United Kingdom, infrastructures that have taken increasing importance in the wake of the Russo-Ukrainian war. Honeywell is not modest about their corporate green credentials while producing more energy. In their promotional material, “Since the Honeywell digital twins can quantify exactly how much energy we are producing at each generating asset and exactly what is being used by each consuming asset, we can do full energy accounting and calculate equivalent CO2 emissions.” The oil platform can supposedly operate more efficiently, with the report claiming a reduction of the equivalent of 1164 automobiles removed from the road while securing European energy in the face of geopolitical instability (Honeywell, 2020). What these systems assume is that two impossible forces can be reconciled through technology whereby limitations to environmental capacity, human life, and resources can be harmonized with an eternal growth of economy.

AI, twinning, and machine learning acquire force in this new managerial-speculative-political ethos. The digital twin platform permits the quantification and management of new increments of geography and velocities of populations and data; this is what “service” denotes. This is also the platformization of infrastructures, as increasingly more and more modes of accessing and leveraging underlying assets are developed and infrastructures (roads, even water or agricultural) are understood as sites to connect various services with clients and of assetization. For example, transit modelers now envision car travel that can be tracked through geographic, but also economic and environmental zones, down to squares of the road, offering credits or penalties depending on routes, and ensuring control over mobility of restricted populations such as immigrants. This is labeled mobility as resource, what was once perhaps considered public a road, is now resourcified through datafication (Waller, 2023; Waller et al., 2024). Similarly, with micro-grids and smart energy grids, energy use can (theoretically) be parsed in new micro-increments and managed in localized environments. Singapore and Tokyo as well as other cities in East Asia regularly add direct-to-consumer marketing capacities into their transit and mobility tracking and modeling systems. Singapore, in particular, has linked its city wide data collection and surveillance with a digital twin for climate resilience, “Virtual Singapore” (Fisch, 2018; Hassoun, 2021; Robles et al., 2023). Evading the need to plan or fully represent environments or futures, twins are a strategy to continually optimize and derive additional value from underlying assets while negotiating intractable tensions such as maintaining the flow of commodities in the face of boundaries and divisions of geopolitics and managing limits to material and environmental health. The key feature is that these technologies are naturalized in everyday use and justified through the discourse of resilience as evidenced by the marketing materials of corporations purveying smart infrastructure services.

**Figure 5.**
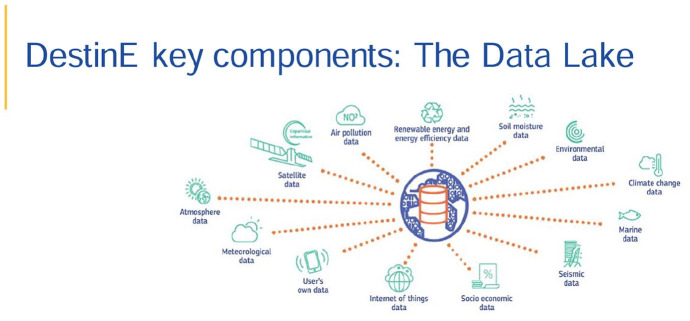
European Union depiction of a data lake in DestinE (Union, 2024).

There are implications for the government for these are also new territories. The imagined data lake (Figure 5), for example, of DestinE extends beyond the boundaries of the EU.

Immigration proponents worry that data from systems such as this might incite anti-immigrant sentiments in forecasting a climate migration upsurge, and that these systems are dual use. The tracking of biodiversity, urban development, and weather patterns, and other data across the world, might also track immigration and refugees; data infrastructures used for purported weather modeling, citizen science, and other projects might serve dual use as military and anti-migrant surveillance systems, particularly since border security and data collected outside of the EU does not subscribe to the AI Act or the General Data Protection Regulations (Eamonn Noonan, 2022). Ecologists worry that remote biodiversity modeling might also result in reductive indicators for biodiversity through standardizing global measures, reducing local measurement, and only evaluating ecosystem health and well-being remotely (Westerlaken, 2024). At the same time, such cross-border data collection might also allow new affiliations and solidarities between scientists and climate activists around the world. The point being that the EU manages both extra-territorial concerns in climate and human population, through projects seemingly located within one space, but monitoring flows of populations and materials across space and transforming these data in simulations, and even into synthetic data sets, the production of what Paulan Korenhof has labeled “synthetic futures” that produce new worlds into the future and direct political action (Edwards, 2010; Korenhof, 2024; Korenhof et al., 2021).

## The generative logic of resilience

There are a few summary points I want to underscore that will come to underpin the new resilient logic of AI (of which twinning is foremost and central as a technique). First, resilience within this genealogy naturalizes uncertainty and volatility as a common perhaps “normal” condition, and consequently the future is recast as “uncertain.” Second, resilience has an “algorithmic” logic in the words of its founders and introduces a set of new practices to model and measure systems. Instead of taxonomy and organizing populations into stable categories, one must define systems in terms of *processes* and measure the relationships *between* populations and potentially other factors (nitrates, carbon, energy, etc.). This orientation turns populations into services; for example, the term “ecosystem services” was part of introducing adaptive management into practice. Today, infrastructure and cloud computing as services and even resources extend this logic and naturalize the assetization of life (Braat and de Groot, 2012). Third, resilience comes with new forms of territoriality, what might be labeled algorithmic territory or “data space.” Fourth, resilience is *generative* in that past data can be used to build concepts but can never actually predict the future. The corollary of such stochastic approaches is that *human* decision-making is flawed. This flaw is an opportunity—for technology (if its AI) or technique—the scenario and now the demo, prototype, experiment, and twin. Finally, in an important divergence from corporate logic, ecologists also emphasized “heterogeneity” and diversity as important to facilitating resilience. Diversity could be an asset or a value. Systems without a surplus of functions and populations could not adapt. Perfectly optimized systems would collapse when change happened. The contemporary corollary might be “just in case” versus “just in time.” But the inverse understanding is that only system operations matter. If a boreal forest continues to be a forest, some species can be allowed to die out or decline, sacrifice, and extinction are normalized and acceptable in the name of system survival; not all connections are as vital to the network.

Perhaps the bleakest statement of the present is a recent ad for twins by the corporation Nvidia:

**The Era of AI-Enabled Digital Twins Has Arrived**

The world’s enterprises are racing to digitalize and become software-defined. With NVIDIA Omniverse™, NVIDIA AI, and OpenUSD, developers are building a new era of digital twins to design, simulate, operate, and optimize their products and production facilities. Once born, these digital twins become the testing grounds for generative physical AI to power autonomous systems. (Nvidia, 2024)

Not only is this era framed as mandating that corporations become “software defined,” a subtle shift from product or service defined. But now twins themselves are alive, they are “born” in this language, but once “born” they become “testing grounds” to build AI. By deduction, then the supply chain is not about building cars or making pharmaceuticals, but rather a food for another technology—mainly AI. And building systems to service technology is a productive and reproductive mandate. This is what is intended by the idea of software or infrastructure as a service. The actual system becomes an asset to derive more value by training new models of AI that themselves can then be monetized and financialized.

## Resilient planet?

The remaining question is are we still in the 1970s? The answer, I argue, is no. The scales and techniques of automation are far greater, and in fact, much depicted here is now encoded into the twins and smart infrastructures managing cities, homes, markets, and factories. The question is how can this history inform the present?

This speculative history of resilience and computing suggests that originally these systems were imagined around a new idea of nature. This model emerged with contradictory demands to maintain carbon-based energy, defuse de-colonial challenges to Western markets and corporations, and manage pollution and biodiversity loss. In our present, resilient ideologies continue to animate the construction, desire, and maintenance of infrastructures of AI and shape geo-politics—a politics that is increasingly about survivalist discourses, catastrophic world making, and battles over resources and technology. Chip Wars, the Inflation Reduction Act, the Belt and Road Initiative, the Digital Silk Road, the CHIPS Act, the EU Commission Green Deal and Digital Strategies all suggest the new territories of contemporary AI politics. And this landscape is only growing in the present.

However, if politics is about power in the present, it is also shaped by imaginaries. While, theoretically, the value ecologists have on diversity could open to a different world model, sadly, the present suggests the opposite. This essay has demonstrated that models of environment and ecosystems are co-produced with technology (new sensor and GPS/GIS systems, digital twins, and so forth) but also that world views and models of nature shape technology and shape the acceptance and adoption of these technologies. Resilience has naturalized ubiquitous computing as necessary for the survival of corporations, cities, and nations. In turn, this evacuation of the social nature of technology permits both corporate actors and the alt-right to embrace survivalist and biologically eugenic discourses while heavily endorsing AI, twinning, and ubiquitous computing as solutions to social problems. Witness billionaire Elon Musk’s statement that Americans must suffer “hardship” to eventually prosper from austerity while supporting increased reproduction for White people, and channeling public funds into his business ventures; all actions met with robust support, rather than critique, in the US election of 2024 (Jones, 2024).

The challenge is to return the social into the techno-natural imaginary of our present. Nvidia and the EU are obviously not the same institutions, nor do they have the same goals. One is a corporation seeking profit. The other is a democratic body responding to an electorate. The question is whether this difference makes a difference in how each produces technological worlds. The resulting contest concerns diversity: diversity of institutions, forms of knowledge, and aesthetics. Seemingly banal and logistical technologies such as digital twins must be recognized for the world making and institution building techniques they are. Such technologies are the sites of political and economic contests of the greatest gravity for the future.

## Notes

1. The global market for digital twins is expected to reach US$259.32 billion in 2030 from US$39.8 billion in 2023. The largest use of twins is in automation of design and manufacturing and enterprise management platforms (Fortune Business Insights, 2024).

2. I took some inspiration from Naomi Klein’s Doppelgänger that posits a paranoid mirror subject as a form of analyzing contemporary reactionary politics (Klein, 2023).

3. Thiel’s famous statement is, “I no longer believe that freedom and democracy are compatible,” later suggesting that one of the great issues in American politics was enfranchising women with the vote in the 1920s. Elon Musk wants the government replaced by “high status males” (Baio, 2024; Reich, 2024).

4. The report stated, “If the present growth trends in world population, industrialization, pollution, food production, and resource depletion continue unchanged, the limits to growth on this planet will be reached sometime within the next one hundred years. The most probable result will be a rather sudden and uncontrollable decline in both population and industrial capacity” (Meadows et al., 1972: 23).

5. Italics mine.

6. Italics mine.

7. Italics mine.

8. Shell had already introduced “Unified Planning Machinery” in 1965, a process for mapping and tracking all operations of the corporation, and projecting future challenges grounded in costs of extraction and use of oil. This was analogous to what many corporations were doing at the time, with total cost analysis, and also included considerable integration of computational and statistical expertise into the planning process Wack (1985a, 1985b).
